# METTL14 Ameliorates Mitochondrial Dysfunction and Autophagy in Lens Epithelial Cells of Diabetic Cataracts via m6A Modification of RPL3

**DOI:** 10.1002/kjm2.70194

**Published:** 2026-05-01

**Authors:** Rui Li, Xue Wang, Wei Du, Qian Li, Hong‐Ping Cui

**Affiliations:** ^1^ Department of Ophthalmology, Shanghai East Hospital, School of Medicine Tongji University Shanghai City China

**Keywords:** autophagy, diabetic cataract, lens epithelial cells, METTL14/RPL3 axis, mitochondrial dysfunction

## Abstract

Diabetic cataracts are a leading cause of blindness, with lens epithelial cells (LECs) exhibiting mitochondrial dysfunction and autophagy inhibition under high glucose (HG) conditions. Methyltransferase‐like 14 (METTL14), an RNA methyltransferase, regulates N6‐methyladenosine (m6A) RNA modification; however, its role in modulating mitochondrial function and autophagy in LECs under diabetic conditions remains poorly understood. This study aims to explore the effects of METTL14 on mitochondrial dysfunction and autophagy in LECs under HG conditions and to investigate the underlying mechanism involving m6A modification of ribosomal protein L3 (RPL3). Primary LECs exposed to HG showed reduced viability, increased reactive oxygen species (ROS), decreased adenosine triphosphate (ATP) levels, and loss of mitochondrial membrane potential (MMP), alongside suppressed autophagy. Knockdown of METTL14 worsened these deficits, while METTL14 overexpression alleviated them. Mechanistically, METTL14 increased m6A modification on RPL3 mRNA, enhancing RPL3 expression. Overexpressing RPL3 improved mitochondrial function, whereas knocking down RPL3 abolished the protective effects of METTL14 overexpression. In diabetic mouse models, adeno‐associated virus (AAV)‐mediated METTL14 overexpression improved lens transparency and reduced oxidative stress, benefits that were reversed by concurrent RPL3 knockdown. In conclusion, METTL14 mitigates HG‐induced mitochondrial dysfunction and autophagy inhibition in LECs by promoting m6A‐dependent upregulation of RPL3, identifying the METTL14/RPL3 axis as a promising target for diabetic cataract therapy.

## 1 Introduction

Diabetic cataract, a common complication in diabetic patients, is characterized by lens opacification leading to progressive vision loss [1]. The pathogenesis involves complex mechanisms, including hyperglycemia, metabolic disturbances, and oxidative stress. Chronic hyperglycemia promotes advanced glycation end product (AGE) accumulation in the lens, altering lens protein structure and function, and inducing mitochondrial dysfunction [2]. Mitochondria, critical for cellular energy metabolism, exhibit impaired function under diabetic conditions, resulting in energy deficiency, excessive reactive oxygen species (ROS) production, and aggravated oxidative stress [3, 4]. These factors collectively contribute to lens epithelial cell (LEC) damage and cataractogenesis. Furthermore, mitochondrial dysfunction interacts with autophagy impairment. Autophagy, an endogenous clearance mechanism for damaged organelles and proteins [5], is frequently suppressed in diabetic conditions, leading to the accumulation of dysfunctional mitochondria and exacerbating oxidative damage in a vicious cycle [6, 7, 8]. Therefore, diabetic cataract progression is closely associated with mitochondrial dysfunction and dysregulated autophagy, which collectively drive cataract formation.

METTL14 (methyltransferase‐like 14), an RNA‐binding protein, plays a pivotal role in N6‐methyladenosine (m6A) modification [9], the most prevalent epitranscriptomic modification in eukaryotic mRNAs, lncRNAs, and noncoding RNAs [10, 11]. As a core component of the m6A methyltransferase complex, METTL14 typically forms a heterodimer with METTL3 to catalyze m6A deposition [12], regulating RNA splicing, stability, and translation [9, 13]. The m6A modification broadly influences gene expression by modulating RNA folding, degradation, and translational efficiency [14]. Emerging evidence implicates METTL14 in diabetic complications: it promotes diabetic nephropathy via m6A‐dependent α‐klotho regulation in glomerular endothelial cells [15], inhibits diabetic cardiomyopathy by suppressing TINCR lncRNA‐mediated pyroptosis [16], and exacerbates oxidative damage and mitochondrial dysfunction in LECs through NEIL1 suppression via the KEAP1/NRF2 pathway [17]. However, the mechanistic role of METTL14 in diabetic cataract pathogenesis remains unclear.

This study aims to investigate how METTL14 regulates mitochondrial function and autophagy in diabetic cataract LECs through m6A‐dependent modulation of ribosomal protein L3 (RPL3) expression. The hypothesis posits that METTL14 improves mitochondrial dysfunction and enhances autophagy via RPL3 m6A modification, thereby mitigating diabetic cataract progression. The findings may provide novel insights into the molecular mechanisms of diabetic cataracts and identify potential therapeutic targets.

## 2 Materials and Methods

### 2.1 Isolation, Culture, and Identification of Primary LECs

Under strict aseptic conditions, C57BL/6 mice were euthanized, and their eyeballs were promptly enucleated. The ocular surface was disinfected with 70% ethanol (Sigma‐Aldrich, USA) and rinsed with ice‐cold phosphate‐buffered saline (PBS; Gibco) to remove debris. The eyeballs were dissected to remove the iris and vitreous body, followed by extraction of the lens. The epithelial tissue covering the anterior capsule of the lens was meticulously isolated under a microscope and digested in pre‐warmed (37°C) enzyme solution containing 0.05% trypsin‐ethylenediaminetetraacetic acid (Trypsin–EDTA; Gibco) and 0.5 mg/mL Collagenase Type I (Sigma‐Aldrich) for 10–15 min at 37°C with gentle pipetting. Digestion was terminated by adding Dulbecco's Modified Eagle Medium/Nutrient Mixture F‐12 medium (Gibco) supplemented with 10% fetal bovine serum (Gibco) and 1% Penicillin–Streptomycin (Gibco). Cells were pelleted by centrifugation (Eppendorf, Germany) for 5 min, resuspended, and seeded in mouse LEC‐specific medium (Shanghai Enzyme‐linked Biotechnology Co. Ltd., China; Cat. No. ml‐CC4442). Cells were cultured at 37°C under 5% CO₂ until reaching 80% confluence, followed by passaging with 0.05% Trypsin–EDTA (Gibco). To simulate diabetic injury, LECs were treated with high glucose (HG, 25 mM). For identification, flow cytometry was performed to assess the positivity of lens‐specific markers (αA‐crystallin, paired box gene 6 [Pax6]).

### 2.2 Cell Transfection

Small interfering RNAs (siRNAs) targeting METTL14 and RPL3, plasmid cytomegalovirus DNA (pcDNA) 3.1 overexpression vectors, and corresponding negative controls were purchased from Invitrogen (China). Following the manufacturer's protocols, these molecular tools were transiently transfected into LECs using Lipofectamine 3000 (Invitrogen, USA). Knockdown or overexpression efficiency was evaluated by quantitative real‐time polymerase chain reaction (RT‐qPCR) and Western blot 48 h post‐transfection.

### 2.3 Cell Counting Kit‐8 (CCK‐8) Assay for Cell Viability

LECs (2 × 10^3^ cells/mL, 100 μL per well) were seeded in a 96‐well microplate, with five replicate wells per group. After overnight culture for cell adhesion, the cells were incubated for 72 h for the CCK‐8 assay. CCK‐8 reagent (10 μL) was added to each well, followed by further incubation for 2 h. Finally, the optical density at 450 nm was measured using a microplate reader.

### 2.4 Lactate Dehydrogenase (LDH) Cytotoxicity Assay

Cellular toxicity was assessed using an LDH assay kit (Beyotime, China) following the manufacturer's protocols. After 18 h of hemoglobin treatment, supernatants were collected and transferred to a 96‐well plate for LDH release measurement. Absorbance at 490 nm was determined using an automated enzyme‐linked immunosorbent assay (ELISA) reader (SpectraMax M5, Molecular Devices, USA).

### 2.5 ROS Detection

Intracellular ROS generation in LECs was quantified employing the oxidation‐sensitive fluorescent indicator 2′,7′‐dichlorodihydrofluorescein diacetate (DCFH‐DA). DCFH‐DA is a cell‐permeable probe that is hydrolyzed intracellularly to DCFH and subsequently oxidized by ROS to yield fluorescent DCF, enabling detection of overall intracellular ROS levels. For the assay, LECs were plated in 6‐well culture dishes at a density of 6 × 10^5^ cells per well. Following experimental treatments, cell suspensions were prepared by enzymatic detachment and subsequent centrifugation. The collected cells were then incubated with 20 μM DCFH‐DA dissolved in PBS for 30 min at 37°C under protected light conditions. After dual washing with ice‐cold PBS to remove excess probe, fluorescence intensity was determined using a flow cytometer (Beckman Coulter, USA) with excitation set at 480 nm.

### 2.6 Adenosine Triphosphate (ATP) Level Measurement

Total ATP content in LECs was determined using a bioluminescent ATP assay kit (Beyotime) according to the manufacturer's protocols.

### 2.7 Mitochondrial Membrane Potential (MMP) Assessment

Changes in mitochondrial membrane potential were evaluated using the 5,5′,6,6′‐tetrachloro‐1,1′,3,3′‐tetraethylbenzimidazolylcarbocyanine iodide (JC‐1) assay kit. Cells (5 × 10^5^ cells/well) were seeded in 6‐well plates, harvested by centrifugation, resuspended in PBS, and stained with JC‐1 at 37°C for 20 min. Stained cells were washed twice with 1 × JC‐1 staining buffer and analyzed immediately by flow cytometry.

### 2.8 Oxidative Stress Markers Detection

The enzymatic activities of antioxidant enzymes, including superoxide dismutase (SOD) and glutathione peroxidase (GSH‐Px), along with the concentration of the lipid peroxidation product malondialdehyde (MDA), were quantitatively analyzed in both cultured LECs and murine lens tissues. Commercial assay kits (Nanjing Jiancheng Bioengineering Institute, China) were employed for these determinations, with all experimental procedures strictly following the manufacturer's protocols.

### 2.9 Monodansylcadaverine (MDC) Staining

The formation of autophagic vesicles in LECs was monitored through MDC labeling (Beyotime, C3019S). Following plating in 6‐well culture dishes, the cells were treated with MDC working solution and maintained at 37°C for 25 min under light‐protected conditions. Subsequent to triple washing with PBS performed in darkness, fluorescent images were captured utilizing an inverted fluorescence microscope (Olympus BX53, Japan).

### 2.10 Western Blot

Total protein from lens tissues and LECs was extracted using radioimmunoprecipitation assay lysis buffer (Beyotime) and quantified via bicinchoninic acid assay. Equal amounts of protein (50 μg) were separated by 12% sodium dodecyl sulfate‐polyacrylamide gel electrophoresis and transferred onto polyvinylidene fluoride membranes. After blocking with 5% skim milk for 1 h at room temperature, membranes were washed three times with tris‐buffered saline with Tween 20 (TBST; 10 min each) and incubated overnight at 4°C with primary antibodies against METTL14, RPL3, bcl‐2 interacting protein beclin‐1 (Beclin‐1), autophagy‐related protein 5 (ATG5), sequestosome‐1 (p62), and β‐actin. Membranes were then washed with TBST and incubated with horseradish peroxidase‐conjugated secondary antibodies (1:3000 dilution) for 1 h at room temperature. Following three additional TBST washes, protein bands were visualized using an enhanced chemiluminescence substrate (Amersham Pharmacia Biotech, UK) and analyzed using Image J software to determine grayscale values.

### 2.11 METTL14 and RPL3 Intracellular Co‐Localization Analysis

To assess co‐localization of METTL14 and RPL3 in LECs, approximately 2000 cells were seeded into 48‐well plates containing coverslips. When cells reached 70%–90% confluence, they were washed three times with PBS, fixed with 4% paraformaldehyde, and permeabilized with 0.5% Triton X‐100 at 4°C for 10 min. After incubation with prehybridization buffer at 37°C for 30 min, cells were hybridized overnight at 37°C in the dark with a Cy3‐labeled RPL3‐specific probe (RiboBio, China). The next day, cells were washed with PBS, retreated again with 0.5% Triton X‐100 at 4°C for 10 min, and incubated overnight at 4°C with anti‐METTL14 primary antibody. Following PBS washes, nuclei were stained with DAPI for 15 min. Co‐localization of METTL14 and RPL3 was visualized using a confocal fluorescence microscope (Carl Zeiss AG, Germany).

### 2.12 Dot‐Blot Analysis

Dot‐blot was performed as previously described. Briefly, total RNA was extracted from LECs using TRIzol. For each sample, 200 ng RNA (1.5 μL) was denatured at 72°C for 5 min, chilled on ice, and spotted onto a nitrocellulose membrane (Amersham, GE Healthcare, USA). After UV crosslinking and blocking, the membrane was probed with an m6A‐specific antibody (202,003, Synaptic Systems). A parallel membrane was stained with methylene blue as an RNA loading control [18].

### 2.13 RNA Immunoprecipitation (RIP) Assay

RIP was performed using the Magna RIP^TM^ RNA‐Binding Protein Immunoprecipitation Kit (Millipore, USA) according to the manufacturer's protocols. Cell lysates (100 μL) were incubated with anti‐METTL14 or immunoglobulin G control antibody‐coated magnetic beads at 4°C for 6 h. After proteinase K digestion to remove proteins, co‐precipitated RNA was extracted and analyzed by RT‐qPCR to quantify RPL3 enrichment (normalized to input). For m6A‐specific RNA binding analysis, RNA fragmented to ~100 nt was immunoprecipitated using the Magna MeRIP^TM^ m6A Kit (Millipore), and m6A‐modified RNA levels were assessed via RT‐qPCR.

### 2.14 Luciferase Reporter Assay

The full‐length RPL3 sequence was cloned downstream of the Firefly luciferase (F‐Luc) coding region in the pmirGLO dual‐luciferase reporter vector (Promega, USA) to construct the wild‐type reporter plasmid (WT‐RPL3). A mutant reporter plasmid (MUT‐RPL3) was generated by substituting adenine with cytosine at the predicted m6A modification sites. Cells in 6‐well plates were transfected for 48 h, and dual‐luciferase activity (F‐Luc and Renilla luciferase [R‐Luc]) was measured using the Dual‐Luciferase Reporter Assay System (Promega). F‐Luc signals represented m6A‐dependent RPL3 expression, while R‐Luc served as an internal control for normalization.

### 2.15 Animal

Male C57BL/6 mice (18–22 g, 6–8 weeks old) were obtained from Hunan Silaike Jingda Experimental Animal Co. Ltd. (China). Mice were housed under a 12‐h light/dark cycle at 22°C ± 2°C with ad libitum access to standard chow and water. All procedures complied with the Guidelines for the Care and Use of Laboratory Animals of Shanghai East Hospital. Animals were randomly divided into control and diabetic nephropathy (DN) groups after one week of acclimatization. DN mice were fed a high‐fat/high‐sucrose diet (HFD; Research Diets Inc., USA) for 4 weeks, followed by daily intraperitoneal injections of streptozotocin (STZ; 80 mg/kg, dissolved in freshly prepared 0.1 M citrate buffer, pH 4.5) for 7 consecutive days. Control mice received normal chow and equivalent citrate buffer injections. After fasting prior to the first injection, mice were allowed to resume feeding. Tail vein blood glucose levels were measured on day 7 post‐STZ initiation. Mice with glucose levels > 11.1 mM (*n* = 64; 6 mice with lower glucose were excluded to equalize group sizes) were randomized into eight subgroups (*n* = 8 mice per group) and maintained on HFD for 8 weeks. This study employed a prevention model design to investigate the protective effects of METTL14 overexpression against diabetes‐induced lens damage. For METTL14 overexpression, oe‐METTL14‐adeno‐associated virus (AAV) or sh‐RPL3‐AAV (100 μL, 1 × 10^13^ GC/mL) and control vectors were administered via tail vein injection three days before STZ treatment to establish gene overexpression or knockdown prior to diabetes induction, followed by biweekly injections to sustain expression. The systemic delivery approach via tail vein injection was selected based on the following considerations: (1) technical maturity and standardized operation ensuring stable and reproducible gene expression; (2) diabetes is fundamentally a systemic metabolic disorder, and systemic gene regulation strategies may more closely approximate potential clinical applications. However, it should be noted that this delivery route results in systemic gene expression, and the observed protective effects on the lens may represent a combination of direct local actions and indirect systemic metabolic improvements. Mice were euthanized via sodium pentobarbital overdose. Eyeballs were enucleated for analysis.

### 2.16 Statistical Analysis

All data are expressed as mean ± standard deviation (SD). Statistical analyses were performed using GraphPad Prism software (version 9.0, GraphPad Software Inc., San Diego, CA, USA). For comparisons between two groups, an unpaired two‐tailed Student's *t*‐test was employed. For comparisons among multiple groups, one‐way analysis of variance (ANOVA) was performed, followed by Tukey's honestly significant difference (HSD) post hoc test for multiple comparisons. P‐value < 0.05 was considered statistically significant. All in vitro experiments were performed with a minimum of three independent biological replicates (*n* = 3), representing three completely independent cell culture and treatment procedures rather than technical replicates of a single experiment. For in vivo experiments, each group contained 8 mice (*n* = 8 mice per group).

## 3 Results

### 3.1 HG Promotes Mitochondrial Dysfunction and Inhibits Autophagy in LECs

**FIGURE 1:**
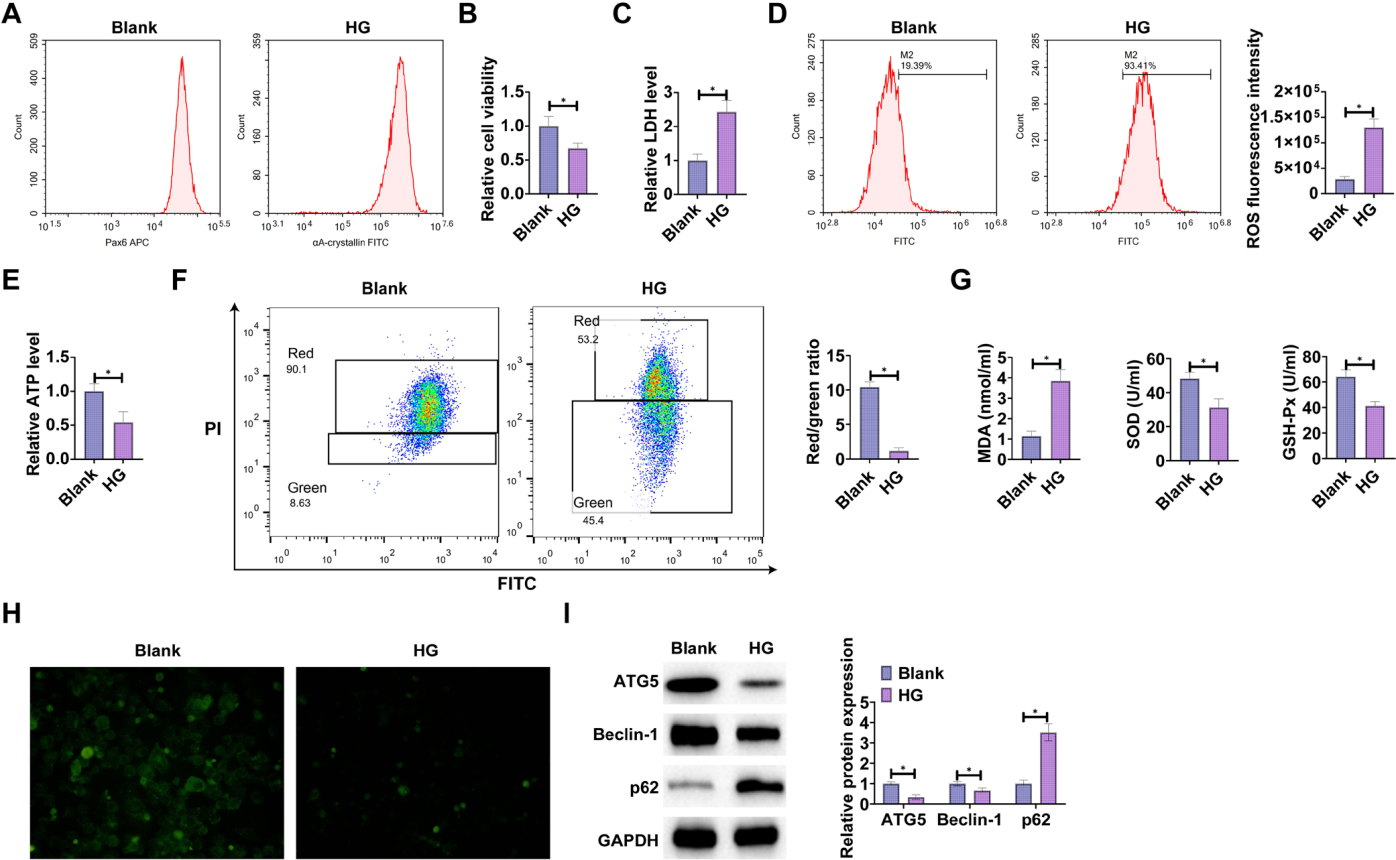
HG Promotes Mitochondrial Dysfunction and Inhibits Autophagy in LECs. Primary LECs were isolated from mice and treated with HG for 12 h. A: Flow cytometry analysis of αA‐crystallin and Pax6 positivity in isolated LECs; B: Cell viability assessed by CCK‐8 assay; C: LDH release measured using commercial assay kits; D: Intracellular ROS levels evaluated using DCFH‐DA probe and flow cytometry; E: ATP content determined with commercial assay kits; F: MMP changes detected by JC‐1 staining; G: SOD, GSH‐PX activities and MDA levels measured using commercial kits; H: Autophagosome formation visualized by MDC staining; I: Protein expression of ATG5, Beclin‐1 and p62 analyzed by western blot. Data are presented as mean ± SD (*n* = 3). * *p* < 0.05.

The study first investigated the effects of HG treatment on mitochondrial function and autophagy activity in LECs. Flow cytometry confirmed successful LEC isolation through positive expression of αA‐crystallin and Pax6 (Figure 1A). CCK‐8 assays revealed significantly reduced cell viability under HG conditions (Figure 1B), while LDH release assays demonstrated increased membrane damage (Figure 1C). ROS levels, assessed using DCFH‐DA staining, were significantly elevated in HG‐treated LECs (Figure 1D). Mitochondrial dysfunction was evidenced by: Decreased ATP levels (Figure 1E). Reduced mitochondrial membrane potential (MMP), measured by JC‐1 staining (Figure 1F). Impaired antioxidant capacity (reduced SOD/GSH‐PX activity and elevated MDA levels) (Figure 1G). Autophagic activity was suppressed under HG conditions, as shown by: Fewer autophagosomes (MDC staining, Figure 1H). Altered expression of autophagy markers (decreased ATG5/Beclin‐1 and increased p62, Figure 1I). These results demonstrate that HG impairs mitochondrial function and suppresses autophagy in LECs.

### 3.2 METTL14 Ameliorates HG‐Induced Mitochondrial Dysfunction and Promotes Autophagy

**FIGURE 2:**
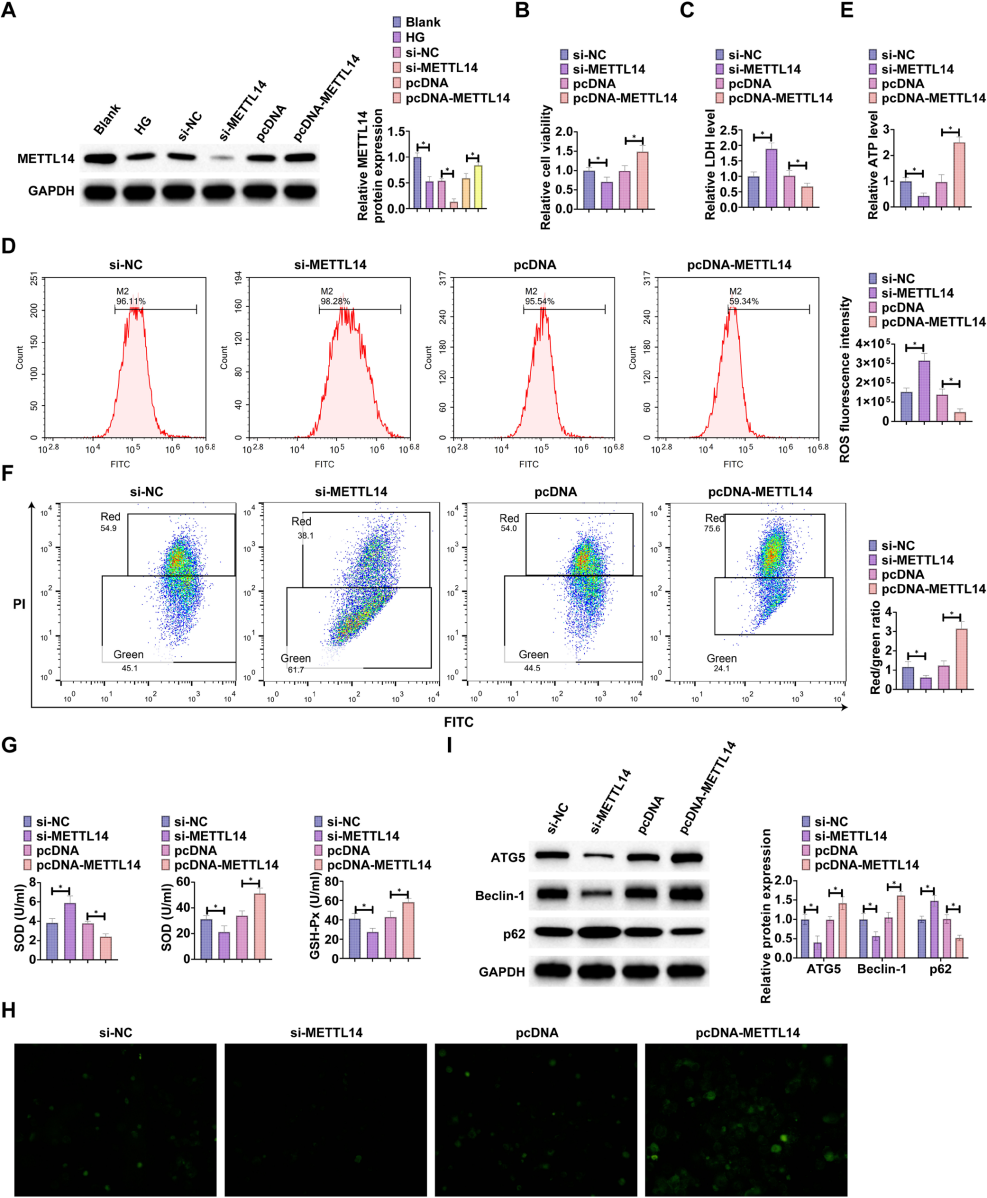
METTL14 Ameliorates HG‐Induced Mitochondrial Dysfunction and Promotes Autophagy. LECs were transfected with METTL14‐targeting siRNA or pcDNA3.1 under HG conditions. A: Western blot analysis of METTL14 protein expression under HG treatment and verification of knockdown/overexpression efficiency; B: Cell viability measured by CCK‐8 assay; C: LDH release quantified using commercial kits; D: Intracellular ROS levels assessed by C11‐BODIPY probe and flow cytometry; E: Cellular ATP content determined with commercial assay kits; F: MMP evaluated by JC‐1 staining; G: Antioxidant enzyme activities (SOD, GSH‐PX) and MDA levels measured using commercial kits; H: Autophagosome formation visualized through MDC staining; I: Expression of autophagy‐related proteins (ATG5, Beclin‐1, and p62) analyzed by western blot. Data presented as mean ± SD (*n* = 3). * *p* < 0.05.

The regulatory role of METTL14 in mitochondrial function and autophagy was investigated in HG‐treated LECs through METTL14‐targeting siRNA knockdown and pcDNA3.1‐METTL14 plasmid overexpression. Western blot confirmed HG‐induced METTL14 downregulation and successful modulation of its expression (Figure 2A). METTL14 knockdown significantly exacerbated HG‐induced cytotoxicity, as shown by reduced cell viability (CCK‐8 assay, Figure 2B) and increased LDH release (Figure 2C), while overexpression attenuated these effects. Oxidative stress analysis revealed that METTL14 knockdown elevated ROS levels (DCFH‐DA staining, Figure 2D), decreased ATP production (Figure 2E), and impaired mitochondrial membrane potential (JC‐1 staining, Figure 2F), whereas overexpression counteracted these changes. METTL14 overexpression also mitigated oxidative damage by preserving SOD/GSH‐PX activity and reducing MDA levels (Figure 2G). Autophagic activity was suppressed by METTL14 knockdown, evidenced by decreased autophagosome formation (MDC staining, Figure 2H) and altered autophagy markers (reduced ATG5/Beclin‐1 and elevated p62, Figure 2I), while METTL14 overexpression restored autophagic flux. These results demonstrate that METTL14 protects against HG‐induced mitochondrial dysfunction and promotes autophagy in LECs.

### 3.3 METTL14 Promotes m6A Modification of RPL3

**FIGURE 3:**
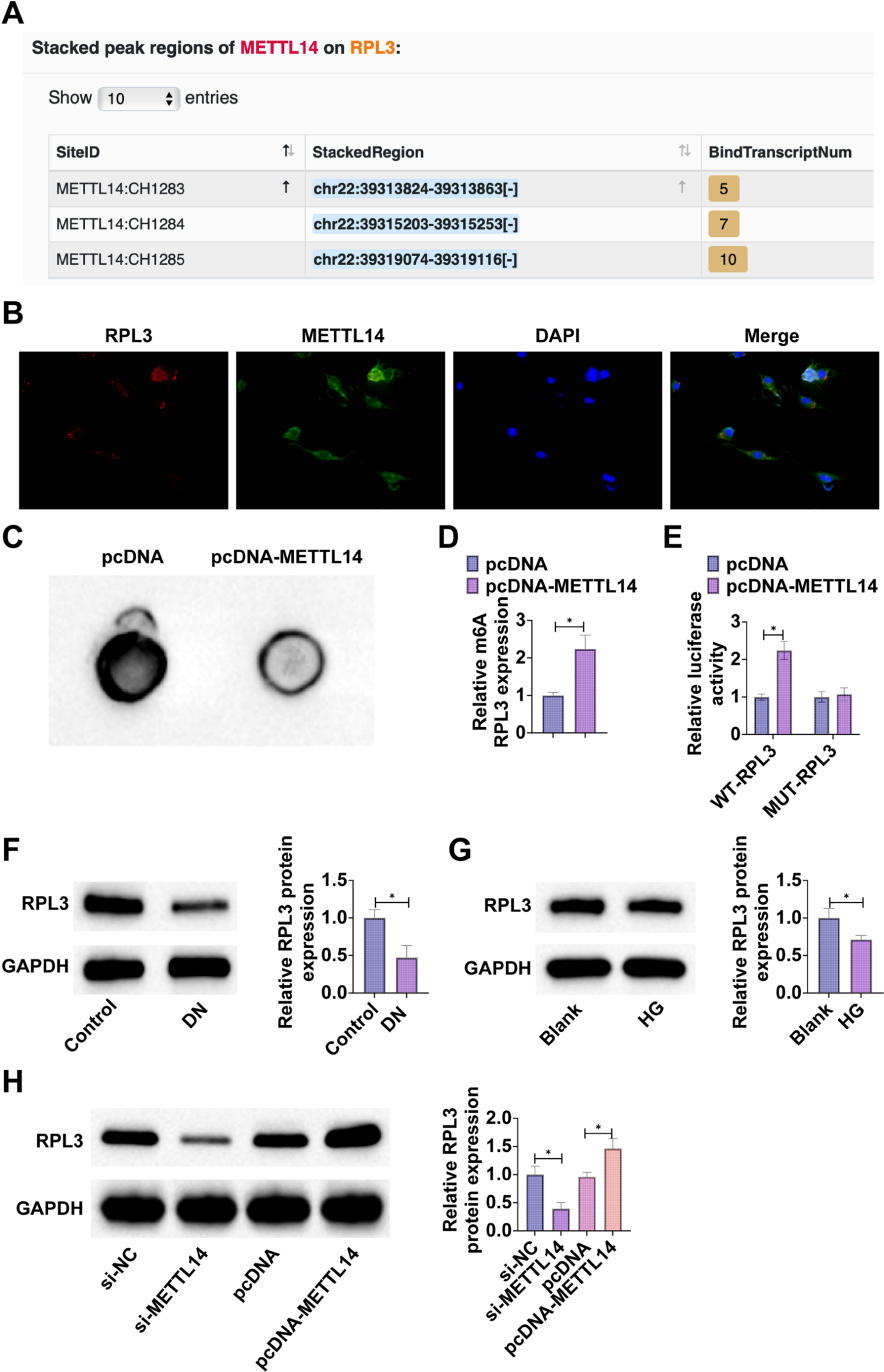
METTL14 Promotes m6A Modification of RPL3. A: Potential binding sites between METTL14 and RPL3 predicted using the Starbase bioinformatics platform; B: FISH analysis confirming cytoplasmic co‐localization of METTL14 and RPL3 in LECs; C: Global m6A levels in METTL14‐knockdown LECs assessed by m6A antibody dot blot, with methylene blue staining as loading control; D: METTL14‐mediated m6A modification of RPL3 detected by MeRIP‐qPCR, showing increased RPL3 m6A modification with METTL14 overexpression; E: Luciferase reporter assay analyzing the effect of METTL14 knockdown on RPL3 transcriptional activity. WT‐RPL3 reporter plasmid contains the complete wild‐type RPL3 sequence cloned downstream of the Firefly luciferase coding region; MUT‐RPL3 reporter plasmid harbors adenine‐to‐cytosine substitutions at predicted m6A modification sites, thereby specifically abolishing m6A modification capacity at these sites to validate the m6A‐dependent regulation of RPL3 by METTL14; F: Western blot analysis of RPL3 expression in normal lens tissues versus diabetic cataract mouse lens tissues; G: Western blot comparison of RPL3 expression between normal and HG‐treated groups; H: Western blot evaluation of RPL3 expression changes following METTL14 knockdown or overexpression. Data presented as mean ± SD (*n* = 3). * *p* < 0.05.

The study investigated METTL14's role in mediating m6A modification in LECs. Bioinformatic analysis (Starbase) predicted potential binding sites between METTL14 and RPL3 (Figure 3A). Fluorescence in situ hybridization confirmed their strong cytoplasmic colocalization in LECs, suggesting a functional interaction (Figure 3B). m6A dot blot assays revealed that METTL14 knockdown significantly reduced global m6A levels (Figure 3C), while MeRIP‐qPCR demonstrated that METTL14 overexpression increased m6A modification on RPL3 (Figure 3D). Luciferase reporter assays showed that METTL14 knockdown decreased transcriptional activity of WT‐RPL3 but not m6A‐site‐MUT‐RPL3, confirming the m6A‐dependent mechanism of METTL14‐mediated RPL3 regulation (Figure 3E). Western blot analysis indicated differential RPL3 expression between normal and diabetic cataract lens tissues, implicating its pathological relevance (Figure 3F). Furthermore, HG treatment downregulated RPL3 in LECs (Figure 3G), and METTL14 modulation directly influenced RPL3 expression—knockdown suppressed, while overexpression enhanced its levels (Figure 3H). These findings demonstrate that METTL14 regulates RPL3 expression via m6A‐dependent mechanisms, highlighting its critical role under HG conditions.

### 3.4 RPL3 Ameliorates HG‐Induced Mitochondrial Dysfunction and Promotes Autophagy

**FIGURE 4:**
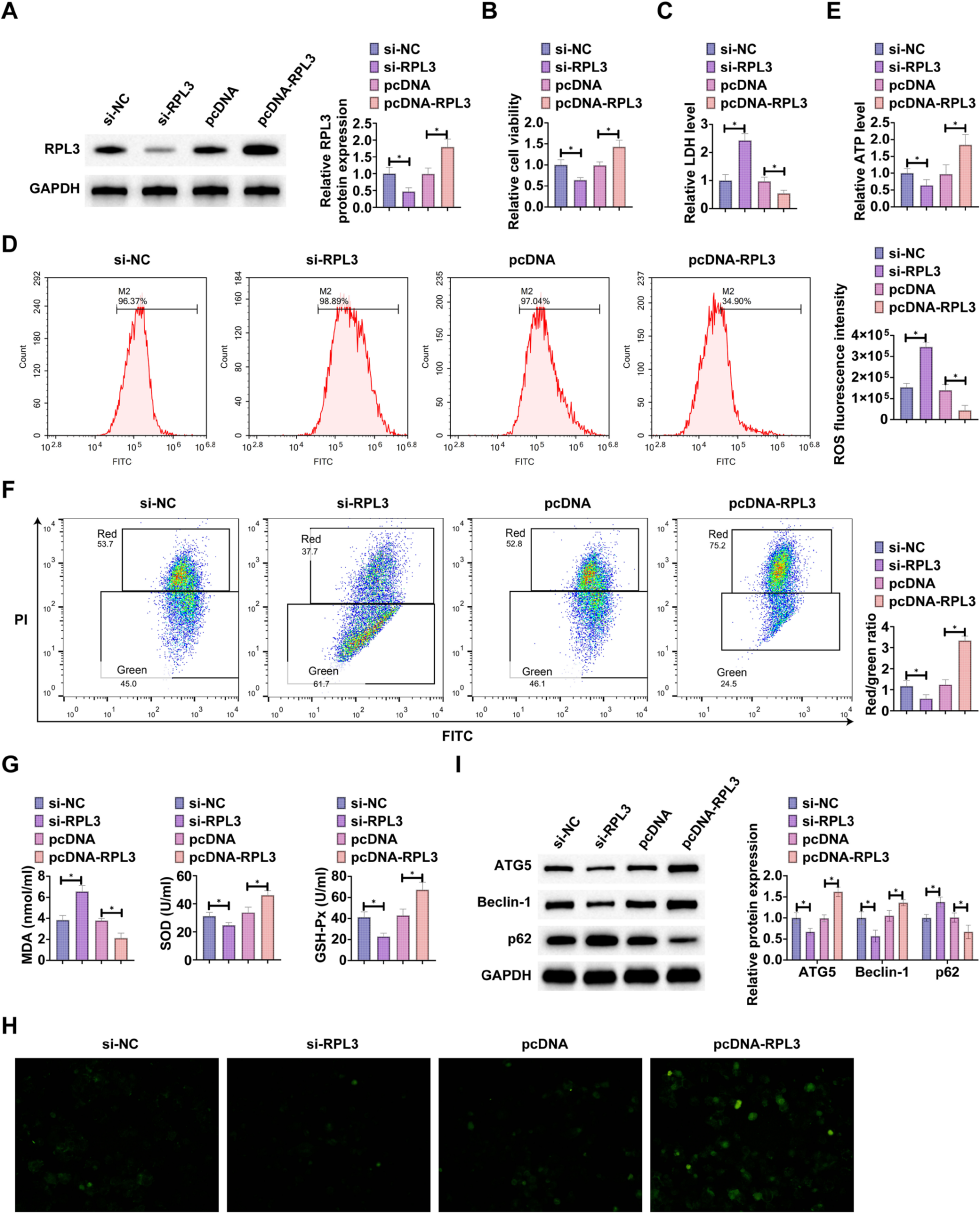
RPL3 Ameliorates HG‐Induced Mitochondrial Dysfunction and Promotes Autophagy. LECs were transfected with RPL3‐targeting siRNA or pcDNA3.1 under HG conditions. A: Western blot verification of RPL3 knockdown and overexpression efficiency; B: Cell viability assessed by CCK‐8 assay; C: LDH release measured using commercial assay kits; D: Intracellular ROS levels determined by C11‐BODIPY probe and flow cytometry; E: Cellular ATP content quantified with commercial kits; F: MMP evaluated via JC‐1 staining; G: Antioxidant enzyme activities (SOD, GSH‐PX) and MDA levels measured using commercial kits; H: Autophagosome formation visualized by MDC staining; I: Expression of autophagy‐related proteins (ATG5, Beclin‐1, and p62) analyzed by western blot. Data are presented as mean ± SD (*n* = 3). * *p* < 0.05.

The study investigated RPL3's role in HG‐induced mitochondrial dysfunction and autophagy regulation in LECs using RPL3‐targeting siRNA knockdown and pcDNA3.1‐RPL3 overexpression. Western blot confirmed successful RPL3 modulation (Figure 4A). RPL3 knockdown significantly reduced cell viability (CCK‐8 assay, Figure 4B) and increased LDH release (Figure 4C), while RPL3 overexpression attenuated these effects. Oxidative stress analysis revealed that RPL3 knockdown elevated ROS levels (DCFH‐DA, Figure 4D), decreased ATP production (Figure 4E), and impaired mitochondrial membrane potential (MMP, JC‐1 staining, Figure 4F), whereas RPL3 overexpression counteracted these changes. RPL3 overexpression also restored antioxidant capacity (increased SOD/GSH‐PX activity and decreased MDA levels, Figure 4G) and enhanced autophagic activity, evidenced by increased autophagosome formation (MDC staining, Figure 4H) and upregulated autophagy markers (ATG5/Beclin‐1) with reduced p62 accumulation (Figure 4I). These results demonstrate that RPL3 protects against HG‐induced damage by improving mitochondrial function, reducing oxidative stress, and promoting autophagy in LECs.

### 3.5 METTL14 Attenuates HG‐Induced Mitochondrial Dysfunction and Promotes Autophagy via Enhancing RPL3 m6A Modification

**FIGURE 5:**
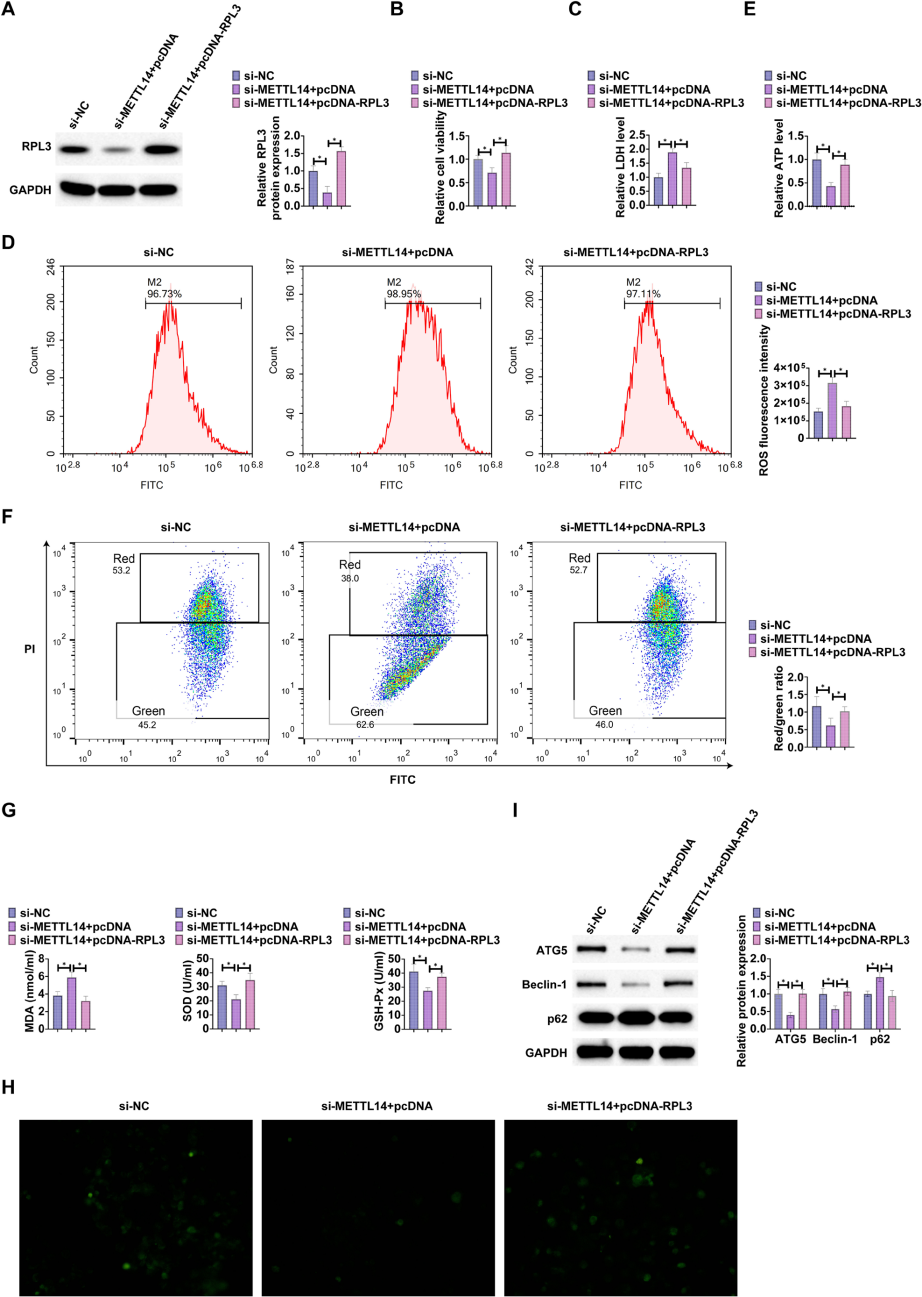
METTL14 Attenuates HG‐Induced Mitochondrial Dysfunction and Promotes Autophagy via Enhancing RPL3 m6A Modification. LECs were co‐transfected with si‐METTL14 and/or pcDNA3.1‐RPL3 under HG conditions to investigate the functional relationship between METTL14 and RPL3. A: RPL3 protein expression analyzed by western blot; B: Cell viability measured using CCK‐8 assay; C: LDH release quantified with commercial assay kits; D: Intracellular ROS levels assessed by C11‐BODIPY probe and flow cytometry; E: Cellular ATP content determined using commercial kits; F: MMP evaluated through JC‐1 staining; G: SOD and GSH‐PX activities along with MDA levels measured using commercial kits; H: Autophagosome formation visualized via MDC staining; I: Protein expression of ATG5, Beclin‐1 and p62 examined by western blot. Data represent mean ± SD (*n* = 3). * *p* < 0.05.

The study examined how METTL14 improves HG‐induced mitochondrial dysfunction and autophagy through RPL3 m6A modification by co‐transfecting si‐METTL14 and pcDNA3.1‐RPL3 in HG‐treated LECs. Western blot confirmed that si‐METTL14 reduced RPL3 expression, while RPL3 overexpression restored its levels (Figure 5A). CCK‐8 assays showed that METTL14 knockdown decreased cell viability, which was partially rescued by RPL3 overexpression (Figure 5B). LDH release assays revealed increased damage with si‐METTL14, attenuated by RPL3 co‐expression (Figure 5C). ROS levels were elevated by METTL14 knockdown but reduced with RPL3 overexpression (DCFH‐DA staining, Figure 5D). ATP production, impaired by si‐METTL14, was restored by RPL3 (Figure 5E). JC‐1 staining demonstrated MMP loss in si‐METTL14‐treated cells, mitigated by RPL3 (Figure 5F). Antioxidant analysis showed si‐METTL14 decreased SOD/GSH‐PX activity and increased MDA, while RPL3 reversed these effects (Figure 5G). MDC staining revealed reduced autophagosomes with si‐METTL14, rescued by RPL3 (Figure 5H). Western blot confirmed METTL14 knockdown downregulated ATG5/Beclin‐1 and upregulated p62, counteracted by RPL3 overexpression (Figure 5I). These findings demonstrate that METTL14 regulates mitochondrial function and autophagy in HG conditions through RPL3 m6A modification.

### 3.6 METTL14 Attenuates Lens Damage in Diabetic Cataract Mice via Enhancing RPL3 m6A Modification

**FIGURE 6:**
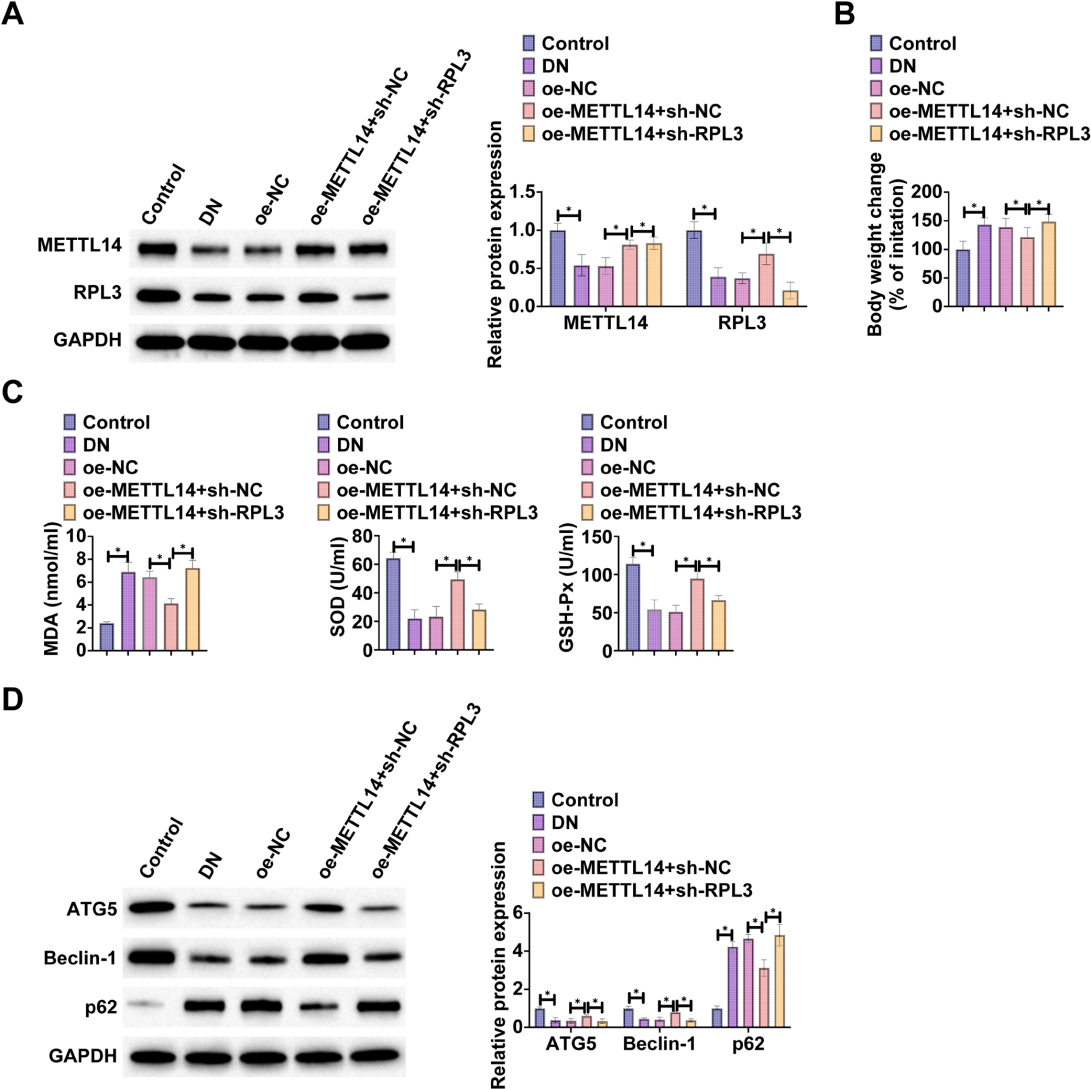
METTL14 Attenuates Lens Damage in Diabetic Cataract Mice via Enhancing RPL3 m6A Modification. A diabetic mouse model was established using STZ injection combined with high‐fat diet feeding. AAV vectors (oe‐METTL14‐AAV, sh‐RPL3‐AAV, or corresponding controls) were administered via tail vein injection three days before STZ treatment (prevention model design). Mice were maintained for 8 weeks after diabetes confirmation before sacrifice. A: Protein expression of METTL14 and RPL3 in lens tissues from different mouse groups analyzed by western blot; B: Body weight measurements across experimental groups; C: SOD activity, MDA content, and GSH‐PX levels in lens tissues; D: Expression of autophagy‐related proteins (ATG5, Beclin‐1, and p62) in lens tissues examined by western blot. Data are presented as mean ± SD (*n* = 8). * *p* < 0.05.

To validate the in vitro findings, a diabetic mouse model was established to investigate METTL14's protective role against diabetes‐induced lens damage. It should be noted that this study employed a prevention model design, wherein AAV vectors were administered three days prior to STZ‐induced diabetes to establish gene overexpression or knockdown before disease onset. Compared to control mice, DN mice exhibited significantly decreased expression of METTL14 and RPL3 proteins in lens tissues (Figure 6A). Tail vein injection of oe‐METTL14‐AAV effectively upregulated the expression of both METTL14 and RPL3, while sh‐RPL3‐AAV injection markedly reduced RPL3 levels. Diabetic mice showed decreased body weight, which was significantly ameliorated by METTL14 overexpression. In contrast, RPL3 knockdown effectively reversed the protective effects of METTL14 overexpression (Figure 6B). Oxidative stress marker analysis confirmed that METTL14 overexpression reduced MDA levels while increasing SOD and GSH‐Px activities in lens tissues—key markers of oxidative stress. These beneficial effects were nullified by RPL3 knockdown (Figure 6C). Western blot analysis demonstrated that METTL14 overexpression enhanced ATG5 and Beclin‐1 expression while decreasing p62 levels, indicating enhanced autophagic flux (Figure 6D). These protein changes were significantly inhibited by RPL3 knockdown. Collectively, the results demonstrate that preventive METTL14 overexpression attenuates diabetic cataract lens damage by promoting RPL3 m6A modification, suggesting that the METTL14/RPL3 axis could be a potential therapeutic target for diabetes‐associated cataracts. However, validation of this pathway's therapeutic efficacy in treating established diabetic cataracts requires further investigation using intervention models initiated after disease onset.

## 4 Discussion

This study elucidates the regulatory roles of METTL14 and RPL3 in LECs under hyperglycemic conditions, particularly in diabetic cataract development. The pathological triad of oxidative stress, mitochondrial dysfunction, and impaired autophagy represents the core mechanisms in diabetic complications, with significant implications for cataractogenesis. The findings demonstrate that METTL14‐mediated m6A modification of RPL3 effectively counteracts these pathological processes, revealing novel therapeutic targets for diabetes‐associated ocular disorders.

As a core component of the m6A methyltransferase complex, METTL14 exhibits cell‐type specificity and spatiotemporal regulation patterns [19]. Previous studies have established its role in modulating RNA translation and degradation, thereby regulating multiple signaling pathways and transcription factor stability to influence cellular growth, differentiation, and stress responses [20, 21, 22]. Under hyperglycemic or oxidative conditions, METTL14 upregulation alters m6A modification patterns, affecting genes involved in metabolic stress, inflammatory responses, and autophagy—processes critical for maintaining cellular homeostasis in both physiological and diabetic states [15, 16]. The current findings demonstrate that METTL14 overexpression enhances the translation of antioxidant, repair, and stress‐response genes, mitigating diabetes‐induced oxidative damage, mitochondrial impairment, and autophagy suppression. Notably, METTL14 exerts protective effects through m6A‐dependent regulation of RPL3, highlighting its therapeutic potential for metabolic disorders.

It is noteworthy that the protective role of METTL14 observed in this study appears to contradict the findings reported by Kang et al. [17], who demonstrated that METTL14 aggravates oxidative damage and mitochondrial dysfunction in LECs. We propose that this apparent discrepancy reflects the inherent complexity and target gene specificity of the m6A modification system, with both studies revealing distinct regulatory facets of METTL14 in LECs from different perspectives. Specifically, the two studies focused on different downstream targets and signaling pathways. Kang et al. investigated how METTL14‐mediated m6A modification suppresses NEIL1 expression, thereby impairing the KEAP1/NRF2 antioxidant pathway, whereas our study identified that METTL14 enhances RPL3 expression through m6A modification, consequently improving mitochondrial function and autophagic activity. As a core component of the m6A methyltransferase complex, METTL14 targets thousands of mRNAs, and the m6A modification of different target genes may produce diametrically opposite downstream effects, either enhancing translation or promoting degradation, depending on the specific reader proteins involved [23, 24]. Furthermore, differences in experimental conditions, including HG concentrations, treatment duration, cell sources, and passage numbers, may also influence the overall phenotypic manifestation of METTL14's effects. This complexity underscores the importance of investigating specific METTL14‐target gene axes and suggests that METTL14 may simultaneously exert both detrimental and protective effects in diabetic LEC injury, with the net outcome determined by the particular target gene network and cellular context.

RPL3, as a ribosomal structural component, plays pivotal roles in protein synthesis during translation initiation and elongation [25, 26, 27]. Beyond its canonical function, RPL3 participates in cell proliferation, stress responses, and metabolic regulation [28, 29]. Emerging evidence implicates RPL3 in diabetes pathogenesis through its roles in maintaining proteostasis, antioxidant capacity, and autophagy [30]. This study reveals that METTL14 enhances RPL3 stability via m6A modification, counteracting hyperglycemia‐induced RPL3 downregulation. RPL3 overexpression improves translational efficiency and metabolic homeostasis, thereby alleviating diabetes‐related cellular damage. Importantly, RPL3 demonstrates unique cytoprotective functions in diabetic ocular models, suggesting potential broad therapeutic applications.

Regarding the mechanistic basis of RPL3's protective effects, we propose that they may be mediated through both canonical and noncanonical pathways. From a canonical perspective, RPL3 upregulation enhances ribosome biogenesis and overall translational capacity, thereby promoting the synthesis of a range of protective proteins, including antioxidant enzymes (SOD, GSH‐Px, catalase), mitochondrial quality control proteins, and autophagy machinery components (ATG protein family, Beclin‐1). Our experimental data indeed demonstrated significantly elevated SOD and GSH‐Px activities, as well as increased ATG5 and Beclin‐1 protein expression in the RPL3 overexpression group, consistent with this hypothesis. Additionally, accumulating evidence indicates that RPL3 possesses extra‐ribosomal functions beyond ribosome assembly, including involvement in cell cycle regulation, p53‐independent apoptosis modulation, and cellular stress responses [28, 29]. Notably, Milenkovic et al. [25] revealed that RPL3 regulates mitochondrial activity through dynamic exchange with RPL3L in cardiomyocytes, which aligns with our observation of RPL3's positive regulatory effect on mitochondrial function in LECs. The precise identification of specific downstream effector proteins of RPL3 represents an important research direction that may require ribosome profiling (Ribo‐seq) or translatomic analyses, which extend beyond the scope of this study.

The mechanistic framework involves multiple aspects: First, m6A modification post‐transcriptionally regulates RNA stability and translational efficiency [31]. METTL14‐mediated m6A enhancement stabilizes RPL3 transcripts, thereby boosting protein synthesis and cellular stress responses. Second, RPL3 upregulation may promote autophagic flux by increasing ribosomal availability for autophagosome biogenesis, which facilitates damaged organelle clearance and the maintenance of cellular homeostasis.

These findings provide novel molecular targets for diabetic ocular complications, particularly cataracts. The METTL14‐RPL3 axis ameliorates key cataractogenic processes, including protein dysregulation, oxidative damage, and autophagic impairment. It should be noted that in our in vivo experimental design, AAV vectors were administered three days prior to STZ‐induced diabetes, constituting a prevention model rather than a therapeutic intervention model. This preventive approach demonstrated that early METTL14/RPL3 pathway augmentation could effectively attenuate subsequent hyperglycemia‐induced lens damage. Additionally, we employed tail vein injection for systemic AAV delivery rather than localized ocular injection. This systemic approach offers advantages including technical maturity, standardized operation, and stable gene expression, while also reflecting the systemic nature of diabetes as a metabolic disorder. However, the amelioration of body weight loss observed in the METTL14 overexpression group suggests significant systemic effects, which may indirectly benefit lens protection through improved overall metabolic status. Our data also confirmed significant alterations in METTL14 and RPL3 expression levels within lens tissues, indicating effective gene manipulation in the target organ. Therefore, the observed lens‐protective effects likely represent a combination of direct local actions and indirect systemic metabolic improvements. Therapeutic strategies targeting METTL14‐mediated RPL3 m6A modification could restore lens transparency through enhanced proteostasis, antioxidant defense, and autophagy. However, the therapeutic efficacy of this pathway for treating established diabetic cataracts requires further validation in studies where intervention is initiated after disease model establishment. Future clinical applications may involve developing small‐molecule modulators of this pathway, with potential extensions to other diabetic complications such as neuropathy and cardiovascular disease.

While this study reveals the potential role of METTL14 and RPL3 in diabetes‐associated ocular disorders, several limitations remain. First, the investigation primarily focused on LECs, while other cell types in different tissues or organs may exhibit distinct responses. Future studies should explore the METTL14/RPL3 axis in other diabetes‐affected tissues, including the retina, nervous system, and heart. Second, although the mechanism by which METTL14 enhances RPL3 function via m6A modification has been elucidated, the precise molecular pathways require further investigation. Key questions remain, including how m6A modification specifically influences RPL3 function—whether through modulating protein–protein interactions or subcellular localization. Third, METTL14 canonically functions as a heterodimer with METTL3 to catalyze m6A deposition, wherein METTL3 provides the S‐adenosylmethionine binding site and catalytic activity while METTL14 primarily mediates RNA substrate recognition and structural stabilization of the complex [32]. Although our data demonstrated that METTL14 overexpression significantly increased RPL3 m6A modification levels, suggesting functional methyltransferase activity presumably involving endogenous METTL3, we did not examine METTL3 expression changes or verify whether METTL14's effects are strictly dependent on METTL3 presence. Future studies employing METTL3 knockdown/overexpression, METTL3‐METTL14 co‐expression, or METTL3 catalytic activity mutants could further elucidate METTL3's role in the METTL14/RPL3 regulatory axis. Fourth, the systemic AAV delivery approach cannot completely exclude contributions from systemic effects (such as overall metabolic improvement or reduced systemic inflammation) to lens protection. Our in vitro experiments on isolated primary LECs validated the direct regulatory effects of the METTL14/RPL3 axis on mitochondrial function and autophagy, supporting a direct protective mechanism. Future studies could employ anterior chamber injection, intravitreal injection, or AAV vectors driven by lens‐specific promoters (such as the αA‐crystallin promoter) to further clarify and quantify the local effects of METTL14 in the lens. Fifth, the precise identification of specific downstream effector proteins synthesized through RPL3‐enhanced translation—which subsequently mediate mitochondrial repair and autophagy activation—warrants investigation using ribosome profiling or translatomic approaches. Additionally, the feasibility and safety of pharmacological or gene‐editing interventions targeting METTL14 and RPL3 require further validation in preclinical studies.

In summary, METTL14‐mediated m6A modification of RPL3 alleviates hyperglycemia‐induced mitochondrial dysfunction and autophagy suppression, significantly mitigating cellular damage. These findings provide novel molecular targets for treating diabetes‐related complications and highlight the therapeutic potential of m6A modification in disease regulation.

## Funding

This work was supported by Young Scientists Fund of Shanghai East Hospital, Tongji University (Grant No. DFPY2024010).

## Ethics Statement

All animal experiments were compliant with the ARRIVE guidelines and were performed in accordance with the National Institutes of Health Guide for the Care and Use of Laboratory Animals. The experiments were approved by the Institutional Animal Care and Use Committee of Shanghai East Hospital.

## Conflicts of Interest

The authors declare no conflicts of interest.

## Data Availability

The data that support the findings of this study are available from the corresponding author upon reasonable request.
